# Phase I Study of Mogamulizumab in Combination with Pembrolizumab in Patients with Relapsed or Refractory Non-Hodgkin Lymphoma—A National Cancer Institute Experimental Therapeutics Clinical Trials Network (NCI-ETCTN) Trial

**DOI:** 10.3390/cancers18020284

**Published:** 2026-01-16

**Authors:** Erel Joffe, Anita Kumar, Joseph M. Tuscano, Alison J. Moskowitz, Colette Owens, Ariela Noy, Maria Lia Palomba, Andrew D. Zelenetz, Andy Ni, Elad Sharon, Santosha Vardhana

**Affiliations:** 1 Memorial Sloan Kettering Cancer Center, Department of Medicine, Lymphoma Service, New York, NY 10065, USAvardhans@mskcc.org (S.V.); 2 Weill Cornell College of Medicine, University of Cornell, New York, NY 10021, USA; 3 School of Medicine, Tel Aviv University, Tel Aviv 6997801, Israel; 4 University of California Davis Comprehensive Cancer Center, Sacramento, CA 95838, USA; 5 The Ohio State University Comprehensive Cancer Center, Columbus, OH 43210, USA; 6 Dana-Farber Cancer Institute, Boston, MA 02215, USA; 7 Cancer Therapy Evaluation Program, National Cancer Institute, Rockville, MD 20850, USA

**Keywords:** pembrolizumab, mogamulizumab, non-Hodgkin lymphoma, hyper-progression

## Abstract

**Introduction**: Immune evasion through inhibition of effector T cells is a key survival mechanism of lymphoma cells. We hypothesized that reinstating effector T cell activity through concurrent inhibition of the PD1/PD-L1 axis and of Treg activity will result in a synergistic anti-tumor effect with an acceptable toxicity profile. **Methods**: Phase I multi-institutional NCI-ETCTN trial aimed to evaluate the safety and tolerability of the combination of mogamulizumab and pembrolizumab in relapsed or refractory non-Hodgkin lymphoma. The study used a 3 + 3 design. Treatment consisted of mogamulizumab 1 mg/kg on days 1, 8, and 15 of cycle 1, followed by 1.5 mg/kg on day 1 of each subsequent 21-day cycle in combination with pembrolizumab 200 mg on day 1 of each cycle. A de-escalation level was defined as a 50% reduction in the dose of mogamulizumab (registered in clinicaltrials.gov NCT03309878). **Results**: The study was discontinued early, after treating seven patients (two angioimmunoblastic T cell lymphoma, four transformed follicular lymphoma, and one diffuse large B cell lymphoma of germinal center subtype) for concerns of futility and non-tolerability. Only two patients completed the first two cycles of treatment. Three patients presented with an early progression and three withdrew consent in the setting of general deterioration with clinically suspected progression. All six patients expired shortly after withdrawal from the study. The remaining patient experienced stress cardiomyopathy during the third cycle and was taken off the study. **Discussion**: In striking difference to the observation in solid malignancies, the combination of mogamulizumab with pembrolizumab was associated with low tolerability and suspected hyper-progression in patients with lymphoma.

## Simple Summary

Lymphoma cells evade immune destruction by suppressing T cells. We hypothesized that combining two T cell enhancing immunotherapies by both blocking the PD1/PD-L1 checkpoint pathway and reducing regulatory T cell (Treg) activity could produce a stronger anti-tumor response. This Phase-I trial tested the safety of combining mogamulizumab (which targets Tregs) with pembrolizumab (a PD-1 inhibitor) in patients with relapsed or refractory non-Hodgkin lymphoma. We treated seven patients on protocol without any clinically meaningful responses and with a very poor tolerability by patients. Only two patients completed the first two treatment cycles. Three experienced rapid disease progression, and three withdrew amid clinical deterioration. Six patients died shortly after leaving the study. The seventh developed stress cardiomyopathy and discontinued treatment. In contrast to findings in solid tumors, this combination showed poor tolerability and may have been associated with hyper-progressi.

## 1. Introduction

The tumor microenvironment (TME) plays key roles in both support and opposition of neoplastic processes, among others, serving as the battleground onto which activated infiltrating T cells (TiL) are recruited to combat malignancy [1,2,3]. One of the core mechanisms by which cancers evade this immune response is activation of inhibitory checkpoint pathways through the programmed cell death protein-1 (PD-1) and its ligand (PD-L1) [4,5,6]. This immune evasion may be enhanced further by chemotaxis and activation of regulatory T cells (Tregs) into the TME [1,2].

Reconstitution of anti-tumor T cell activity by inhibition of the PD-1/PD-L1 pathway has had a transformative role in the treatment of Hodgkin’s lymphoma [7,8,9]. However, the activity of these agents in non-Hodgkin’s lymphoma (NHL) has been disappointing [10,11]. A possible mechanism of immune evasion in these cases is PD-1 inhibition on Treg leading to their expansion and activation [10]. In solid malignancies, several studies have demonstrated an inverse correlation between the extent of CD8+ TiL and the extent of FoxP3+ Treg TiL with prognosis [2]. Furthermore, in preclinical and clinical studies, it has been demonstrated that tumor trafficking of Treg, mediated by CC chemokine receptor 4 (CCR4), is upregulated in tumors on checkpoint inhibitors treatment [12,13,14]. We, therefore, hypothesized that inhibition of Treg trafficking by CCR4 using mogamulizumab in combination with PD-1/PD-L1-directed therapy with pembrolizumab will have a synergistic effect that would bypass resistance to checkpoint inhibition in NHL. A similar hypothesis has been pursued concurrently in solid malignancies [15].

## 2. Materials and Methods

This was an investigator-initiated open-label phase I multicenter study sponsored by the National Cancer Institute Experimental Therapeutics Clinical Trials Network (NCI-ETCTN). The primary objective was to evaluate the safety and tolerability of mogamulizumab (KW-0761) when administered in combination with pembrolizumab (MK-3475) in patients with relapsed or refractory NHL. The secondary objective was to observe and record anti-tumor activity. The protocol had a planned expansion to a phase II portion but was discontinued early. The study was approved by the NCI-ETCTN central review board as well as the review boards at participating institutions and registered at clinicaltrials.gov (NCT03309878, registered on 16 October 2017). The study was conducted in accordance with the guidelines of Good Clinical Practice and the Declaration of Helsinki.

### 2.1. Patients and Treatment

Adult patients (age ≥ 18 years) were included with histologically confirmed, measurable, relapsed or refractory (R/R) non-Hodgkin’s lymphoma (NHL) for which no additional standard or approved therapies were available. Patients had to have an Eastern Cooperative Oncology Group (ECOG) performance status ≤ 2 and have adequate organ and bone marrow function. Patients with active cerebral or meningeal involvement by lymphoma were excluded, as were patients with an active autoimmune disease requiring systemic treatment or a history of pneumonitis. Also excluded were patients who have had a prior allogeneic hematopoietic stem cell transplant (HSCT) or in whom an HSCT was a plausible treatment option in the immediate future.

The study used a standard 3 + 3 design to identify the maximum tolerated dose, or the highest protocol-defined dose, as the recommended phase II dose (RP2D) based on dose-limiting toxicity (DLT). Patients who did not complete the safety follow-up, spanning two treatment cycles, for reasons other than the occurrence of a DLT were replaced. Treatment was administered on a 21-day cycle.

Two dose levels were planned. The initial dose was defined as mogamulizumab intravenously (IV) 1 mg/kg over at least one hour on days 1, 8, and 15 of cycle 1, followed by 1.5 mg/kg on day 1 of each subsequent 21-day cycle. This dose and schedule were designed to align with the dosing schedule of pembrolizumab. Accordingly, the dose of mogamulizumab was increased to 1.5 mg/kg (as opposed to the standard recommended dose of 1 mg/kg every 14 days) based on the dose administered in a development program in T cell lymphomas [16]. A flat dose of 200 mg of IV pembrolizumab was administered on day 1 of each cycle based on the standard dosing per the Food and Drug Administration label [17]. Doses could be interrupted, delayed, or stopped depending on tolerability. A de-escalation level, limited to reduction of mogamulizumab, was defined as 0.5 mg/kg weekly for three weeks followed by 1 mg/kg once every 21 days. After treating the first 3 patients, we transitioned to the de-escalation dose due to concerns of tolerability. Premedication against infusion reactions consisted of oral acetaminophen 650 mg, IV diphenhydramine 50 mg (or equivalent), and IV hydrocortisone 50 mg at least 30 min before the first and second doses of mogamulizumab. In patients who experienced an infusion reaction, the protocol recommended continuing premedication. Treatment was planned to be given until progression, intolerable adverse events, and no longer than 24 months. Concurrent administration of other anti-neoplastic drugs or any immunosuppressive treatment was prohibited (unless to control treatment-related adverse events).

### 2.2. Toxicity and Response Assessments

Adverse events were recorded and graded according to Common Terminology Criteria for Adverse Events v5.0. The DLT window was defined as the first 2 cycles of treatment (6 weeks) and included any grade 4 hematological toxicity (except lymphopenia) lasting ≥7 days; any grade 3 or higher clinical non-hematological toxicity of any duration; or any grade 3 or higher laboratory non-hematological toxicity lasting ≥72 h and requiring medical intervention. Safety was analyzed for all patients who had received at least one dose (even if a partial dose) of mogamulizumab in combination with pembrolizumab.

The response assessment was performed according to the Lugano Classification for response assessment in lymphoma. Indeterminate response (IR) was defined as any progression during the first 12 weeks of therapy or the appearance at any time of new lesions or growth of existing lesions occurring in the context of lack of overall progression as measured by the sum of the product of the diameters (SPD) of up to six target lesions [18]. In cases with IR, the investigator could opt to continue treatment and re-image after an additional 4 weeks [19].

### 2.3. Statistical Analysis

The sample size for phase I was based on a standard 3 + 3 dose-finding design, and with two dose levels under consideration, a maximum of 12 patients was planned (3–6 patients/cohort). A phase II portion was planned aimed at comparing the combination of mogamulizumab and pembrolizumab with pembrolizumab monotherapy on a 1:1 ratio. This portion of the study assumed progression-free survival at 12 months of 30% and 10% in the combination versus monotherapy, respectively.

Demographic and baseline characteristics and efficacy and safety endpoints are summarized descriptively. Categorical values are presented as frequency and percentages, and continuous variables as median and range.

## 3. Results

Eight patients were enrolled in the study, of whom seven received the first dose of mogamulizumab and pembrolizumab (one patient deteriorated and died shortly after enrollment). There were two patients with R/R angioimmunoblastic T cell lymphoma; four had transformed diffuse large B cell lymphoma (DLBCL) from a background of follicular lymphoma (tFL), and one had germinal center subtype (GCB) DLBCL. All patients were male, with a median age of 65 (range 55–77); five had an ECOG of 0–1 and two an ECOG of 2. All patients had a stage IV disease, and LDH was elevated above the upper limit of normal in all but one. The median number of prior lines of therapy was six (2–9), and four patients with DLBCL/tFL had prior treatment with CAR-T (Table 1). The first three patients received treatment on the initial dose level (mogamulizumab 1 mg/kg), which was then decreased to dose level -1 (mogamulizumab 0.5 mg/kg) due to concerns of low tolerability.

**Figure 1:**
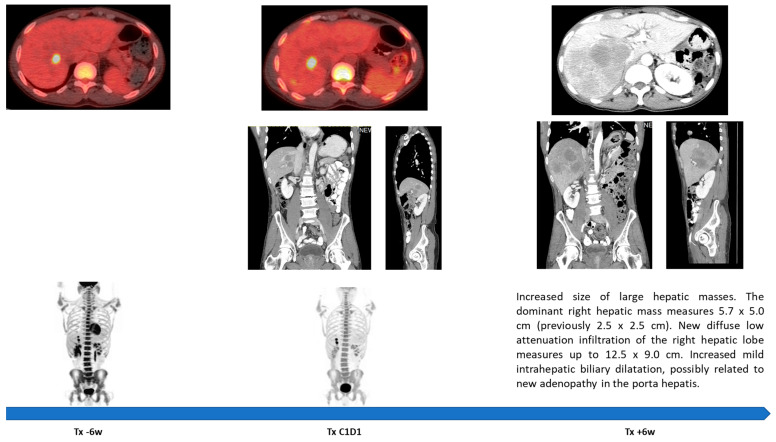
Subject NY016-0008 hyper-progression noted 6 w from initiation of therapy. Note the limited progression “off-treatment” between 6 weeks prior to initiation of study drugs (Tx − 6w) as compared to the extent of progression during the 6 weeks following initiation of study drugs (Tx + 6w).

Only four patients completed all three infusions in cycle 1, of whom only two completed cycle 2 of treatment. There were no treatment-related DLTs; however, patients reported anorexia and/or fatigue (Table 2). Three patients were taken off the study for an early progression of disease (POD; Figure 1), and three withdrew consent due to fatigue and general deterioration. These patients appeared clinically to have progressed, but all opted for comfort care and did not pursue further imaging or treatment. There were no dermatologic adverse events. One patient (AITL) experienced a DLT shortly after C2D1, presenting with grade-4 hypercalcemia and acute kidney injury. These were considered by the treating team to be unrelated to the study drug and attributed to POD. One patient (AITL) continued to cycle 3 with an indeterminate response and experienced a transfusion reaction followed by stress cardiomyopathy (Takotsubo). He was taken off the study and was subsequently also found to have a POD. The stress cardiomyopathy had resolved over the ensuing months with a return to baseline ejection fraction, though with a small residual area of apical hypokinesis. All but this last patient had died shortly after exiting the study.

## 4. Discussion

This phase I multi-institutional NCI-ETCTN trial aimed to evaluate the safety and tolerability of the combination of mogamulizumab and pembrolizumab in R/R NHL. The governing hypothesis was that reinstating effector T cell activity through the PD1/PD-L1 axis concurrently with inhibition of Treg activity will result in an additive, if not, synergistic, anti-tumor effect with an acceptable toxicity profile. The study was halted early after three patients presented with rapid progression, another three withdrew consent in the setting of general deterioration and clinically suspected progression, and a single patient with a mixed response (later confirmed as a progression) experienced stress cardiomyopathy (Takotsubu) shortly past the DLT window.

The combination of mogamulizumab with nivolumab (checkpoint inhibitor) was recently evaluated in a phase I/II trial in patients with R/R solid malignancies. The study, which included 114 patients, was discontinued due to futility (ORR 11%; PD 62%; responses primarily in hepatocellular carcinoma ORR 17% and ovarian carcinoma ORR 14% with negligible responses in colon and pancreatic carcinomas) [15]. However, there were no DLTs noted for a treatment with a similar dose intensity (1 mg/kg of mogamulizumab combined with the standard dose of nivolumab). Importantly, in striking contrast to our cohort, fatigue/asthenia were the cause for treatment discontinuation in less than 5% (five patients). In that study the most common serious treatment-related adverse events were autoimmune colitis (4%), diarrhea, infusion-related reaction, and drug eruption rash (each 3%), and erythema multiforme and thrombocytopenia (each 2%) [15].

Unfortunately, six out of seven patients included in this study did not complete the 6-week safety period and thus were not evaluable for response nor for correlative lab and biopsy analyses planned for the first day of cycle 3. In three patients there was a confirmed progression of disease, while three withdrew consent without further evaluation. In the latter, the clinical picture was highly suggestive of progressing disease. Considering the reported good tolerability of the combination of mogamulizumab with nivolumab in solid malignancies, and the similar rapid deterioration noted in patients treated on the reduced dose level, it is plausible that all patients had experienced a POD as the cause for their decline. Indeed, all but one patient expired shortly after being taken off the study. This observation suggests the combination of mogamulizumab with pembrolizumab may have had a paradoxical effect of promoting tumor progression in patients with AITL and DLBCL-GCB/tFL. Arguably, the possibility of cytokine-release syndrome (CRS), immune effector cells-associated hemophagocytic lymphohistiocytosis (IEC-HLH)-like syndrome, or a pseudo-progression due to an inflammatory intra-tumoral effect is less likely, as no patient displayed symptoms meeting the diagnostic criteria of CRS/IEC-HLH, and all but one succumbed to their disease shortly after discontinuation of trial treatment.

Checkpoint inhibition with nivolumab has been recently reported in association with hyper-progression in three patients with adult T cell leukemia/lymphoma (ATLL) [20]. Biomolecular analysis of pre/post treatment from these patients demonstrated similarities in gene expression among the ATLL cells and different Treg-like subtypes (expressing higher levels of CTLA4, LAG-3, and OX40), supporting a hypothesis that PD-1 blockade in Treg cells results in their expansion and promotes tumor growth in ATLL even in the presence of potent antitumor effector T cell immune responses [20,21]. The addition of mogamulizumab was anticipated to counter this hypothesized pembrolizumab-induced Treg expansion, and thus our observation of suspected hyper-progression is intriguing. A possible explanation is loss of inhibition of T follicular helpers (T_FH_). T_FH_ are known to play a key role in the support of proliferation in germinal center lymphomas and are the cell of origin of AITL [12,22]. These cells are known to be inhibited by a subset of follicular regulatory T cells, which may have been abrogated by the concurrent administration of mogamulizumab [23]. In addition, it seems that T_FH_ possess both supporting and inhibitory properties, the sum of which determines their net effect on germinal center proliferation. Thus, further research is necessary to elucidate the intricate combinatory effect of cytotoxic, regulatory and helper T cells in the immune microenvironment of germinal center lymphomas.

## 5. Conclusions

Mogamulizumab in combination with pembrolizumab was associated with low tolerability in patients with lymphoma and may be associated with hyper-progression by biomolecular mechanisms yet unclear.

## Figures and Tables

**Table 1:** Patient characteristics.

Index	Histology	Age	Sex	ECOG	Prior Lines	Hx CAR-T	Stage	Extranodal	LDH	IPI	Mogamulizumab	Pembrolizumab	Last Infusion	Off Study	Comments
1	AITL	55	M	0–1	2	0	4	0–1	324	3	1	200	C2D1	Withdrawal	Hypercalcemia and AKI
2	AITL	71	M	0–1	9	0	4	0–1	304		1	200	C3D1	SAE	Stress cardiomyopathy
3	tFL	72	M	0–1	8	1	4	>1	450	4	1	200	C1D8	Withdrawal	General deterioration
5	tFL	77	M	2	6	1	4	>1	315	5	0.5	200	C1D8	Withdrawal	General deterioration
6	tFL	58	M	2	2	0	4	>1	1343	3	0.5	200	C1D15	POD	
7	tFL	53	M	0–1	5	1	4	>1	461	3	0.5	200	C1D1	Death	
8	DLBCL-GCB	50	M	0–1	6	1	4	0–1	237	2	0.5	200	C1D15	POD	

AITL—angioimmunobalstic T cell lymphoma; DLBCL-GCB—diffuse large B cell lymphoma of germinal center like subtyp; Hx CAR-T—prior treatment with chimeric antigen receptor modified T cells; IPI—international prognostic index; M—male; POD—progression of disease; SAE—serious adverse event; tFL—transformed DLBCL from follicular lymphoma.

**Table 2:** Adverse events (occurring in >1 patient).

AE/Grade	All	1	2	3	4
Anorexia	5	1	4		
Vomiting	4	4			
Fatigue	3	1	2		
Dehydration	3		3		
Hyponatremia	3	2		1	
Fever	3	1	2		
Confusion	2	1	1		
Fall	2	1	1		
Weight Loss	2	1	1		
Diarrhea	2		2		
Acute Kidney Injury	2			2	
Cough	2		2		
Hypotension	2		1		1
Urinary Tract Obstruction	2			2	
Anemia	2	1			1
Platelet Count Decreased	2	1	1		

Adverse events by frequency. Five of seven patients experienced various manifestations of general deterioration including anorexia, mild vomiting, dehydration, mild confusion and fatigue.

## Data Availability

Trial data are unavailable due to privacy restrictions.

## Abbreviations

AITL	angioimmunoblastic T cell lymphoma
ATLL	adult T cell leukemia/lymphoma
CCR4	CC chemokine receptor 4
CRS	cytokine-release syndrome
DLBCL	diffuse large B cell lymphoma
GCB	germinal center subtype
IR	indeterminate response
IV	intravenously
NCI-ETCTN	National Cancer Institute Experimental Therapeutics Clinical Trials Network
NHL	non-Hodgkin’s lymphoma
ORR	overall response rate
PD	progressive disease
PD-1	programmed cell death protein-1
PD-L1	programmed cell death ligand-1
POD	progression of disease
R/R	relapsed or refractory
RP2D	recommended phase II dose
T_FH_	T follicular helpers
tFL	transformed diffuse large B cell lymphoma from a background of follicular lymphoma
TiL	tumor infiltrating T cells
TME	tumor microenvironment
